# Answer to the letter from Joanna Zakrzewska

**DOI:** 10.1007/s10194-011-0411-8

**Published:** 2011-12-31

**Authors:** Jes Olesen

**Affiliations:** Department of Neurology, Danish Headache Center, University of Copenhagen, Glostrup, Denmark

This letter raises many important issues. First of all, why are headaches different from other chronic pain disorders [1]? This is partly historic because headaches are being cared for by neurologists and other pains by other specialists. There are, however, a few other important reasons.

Some of the most important headache disorders respond to treatments that have absolutely no effect on pains in any other part of the body or of any other aetiology. This is true for migraine, cluster headache and a number of other primary headaches. When it comes to secondary headaches this is also true and here of course neurological expertise is required. Furthermore, daily dosing of analgesics in patients with primary headaches will often lead to a worsening of the situation, so-called medication overuse headache. Finally, headaches are about as common as all other kinds of pains taken together and have enormous socioeconomic costs. Headache by itself is a fairly large field with thousands of doctors involved in its treatment and the field has a long tradition for annual congresses with significant participation and full programmes. Increasingly, it is realized that the trigeminal system is different from the spinal cord pain perceiving system. A number of compounds cause headache after intravenous infusion but not pain in any other part of the body.

That having been said, there are many commonalities.

Ten years ago, upon my initiative, the IASP Congress and the Congress of the International Headache Society in Paris were placed in succession. Few people participated in both congresses—I myself being one of them. Those who did realized that it is impossible to participate in both congresses because it means 7 days of intense congress activity. Multidisciplinary clinics have been a mainstay in the treatment of chronic pain for decades but only in recent years has this concept been applied to headache. While initial results of these clinics are promising, it must be confessed that hard scientific evidence, such as derived from randomized studies, remains relatively weak. This is not to say that these clinics should not continue and further develop their treatments, but it is hard to say that there is a lot to learn from each other when it comes to multidisciplinary treatment.

In summary, there are very good reasons for these two fields to remain separated but common fields of interest should be defined and studied. It seems intuitively obvious that there could be much to gain if joint task forces were formed to analyse this spectrum of possibilities.
